# Awareness of Hypertension, Hypercholesterolemia, and Diabetes Mellitus and Associated Characteristics in Russian Adults

**DOI:** 10.1155/2024/8542671

**Published:** 2024-03-25

**Authors:** Filip Sahatqija, Monica Hunsberger, Sarah Cook, Kamila Kholmatova, Marina Shapkina, Sofia Malyutina, Alexander V. Kudryavtsev

**Affiliations:** ^1^Shalgrenska Academy, University of Gothenburg, Gothenburg 41390, Sweden; ^2^School of Public Health, Faculty of Medicine, Imperial College London, London SW7 2AZ, UK; ^3^International Research Competence Centre, Northern State Medical University, Arkhangelsk 163069, Russia; ^4^Department of Community Medicine, UiT The Arctic University of Norway, Tromsø N-9037, Norway; ^5^Research Institute of Internal and Preventive Medicine, Branch of Institute of Cytology and Genetics, Siberian Branch of the Russian Academy of Sciences, Novosibirsk 630089, Russia; ^6^Department of Therapy, Haematology and Transfusiology, Novosibirsk State Medical University, Novosibirsk 630090, Russia

## Abstract

Russia has higher cardiovascular disease (CVD) mortality compared to other European countries. The major CVD risk factors are age, male sex, and three conditions, namely hypertension, hypercholesterolemia, and diabetes mellitus (DM). This study aimed to assess awareness of these three conditions among Russian adults (*N* = 3803) and the associated socio-demographic, lifestyle, and health characteristics. We used cross-sectional data from a randomly drawn population-based sample of Russians aged 35–69 years, who participated in the Know Your Heart (KYH) study conducted in Arkhangelsk and Novosibirsk between 2015–2018. Participants' self-reported awareness of hypertension, hypercholesterolemia, and DM was assessed against the measures at the KYH health check (blood pressure, cholesterol, HbA1c and/or use of medication for each condition). Prevalence estimates for the awareness were age- and sex-standardized to the Standard European Population. Socio-demographic, lifestyle, and health-related correlates of the awareness were investigated using logistic regression modelling. Among participants with hypertension (*N* = 2206), hypercholesterolemia (*N* = 3171), and DM (*N* = 329) recorded at a health check, 79%, 45%, and 61% self-reported these conditions, respectively. Higher awareness of hypercholesterolemia and hypertension was associated with older age, female sex, nonsmoking status, obesity, and history of CVD diagnoses. Low household income and history of CVD diagnoses were associated with being aware of DM. The awareness rates of hypertension were relatively high, whereas awareness rates of hypercholesterolemia and DM were relatively low. CVD prevention and early intervention could be improved in Russia through increasing the awareness of the risk factors.

## 1. Introduction

Cardiovascular diseases (CVDs), a cluster of disorders of heart and blood vessels, are the leading cause of death globally [1]. However, many countries have reduced CVD incidence and mortality through the successful implementation of prevention programmes and the introduction of surgical treatment strategies [2]. Eastern European countries fall behind other European countries in reducing CVD mortality rates [3]. In 2019, the lowest age-standardized CVD mortality rate of 190 per 100,000 population was registered in France [4]. For comparison, Russia had a CVD mortality rate of 574 per 100,000 population in 2019 [5], which was the third highest in Europe after Ukraine and Bulgaria [6]. Despite existing research, the causes of the high CVD mortality rates in Russia are still not extensively explained [7, 8].

Hypertension, hypercholesterolemia, and diabetes mellitus (DM) are the major CVD risk factors [9, 10]. At the early stages, these conditions are often latent due to their asymptomatic or mild manifestations [11–13], and individuals may be unaware of these conditions until they progress to exhibiting symptoms or complications [14]. Public health interventions aimed at raising awareness and early diagnosis of modifiable risk factors are acknowledged tools to reduce CVD morbidity and mortality [14]. To be effective, campaigns must be population-specific and culturally appropriate to target the high-risk groups [15].

Previous research has reported associations between the awareness of hypertension, DM, and hypercholesterolemia and sociodemographic factors (age, sex, education level, employment status, place of residence, and income) [3, 16, 17]. Other authors also described associations of the awareness with behavioural factors such as physical activity, smoking, alcohol consumption, obesity, and previous experience with cardiovascular events or comorbidities [18–20].

Awareness of hypertension in Russia was previously estimated to be 68-80% in males and 76-86% in females [3, 21–24]. Factors associated with hypertension awareness were older age [21–23], female sex [3, 21, 23], higher level of education among males [21], and urban residence [21]. The HAPIEE study (2002–2005) showed relatively low awareness of diabetes (30.8% in males and 45.2% in females) and hypercholesterolemia (11.2% in males and 15.3% in females) in Russia compared with Poland, Lithuania, and the Czech Republic [3]. Several studies assessed the control of DM and high cholesterol [25–27], but we could not find Russian population-based studies focusing behavioral and health characteristics associated with the awareness.

This study aimed to assess awareness of hypertension, hypercholesterolemia, and DM in Russian adults with objective confirmation of these conditions and to investigate socio-demographic, lifestyle, and health-related correlates of awareness.

## 2. Materials and Methods

### 2.1. Study Design

The study is based on data from the “Know Your Heart” (KYH) cross-sectional study, conducted from 2015–2018 in two Russian cities, Arkhangelsk and Novosibirsk, with a general population sample aged 35–69 years.

### 2.2. Recruitment of Participants

The study's sampling frame was drawn from the databases of the Arkhangelsk and Novosibirsk regional health insurance funds (lists of depersonalized addresses of residents with mandatory health insurance supplemented by age and sex). Random addresses were selected for home visits, stratified by age and sex, targeting to recruit equal numbers of participants by sex and 5-year age groups. Trained interviewers visited the selected addresses and invited household members of the predefined age (±2 years) and sex to participate in the study, one person per household. After the ascertainment of informed consent, a participant underwent a baseline interview at home. After the interview, participants were invited to a health check at a polyclinic. The health check comprised a physical examination of cardiovascular health and a medical interview, which included the collection of self-reported data on health status and medication use. Details of the KYH study rationale, design, sampling, and data collection procedures have been published previously [28].

The response for the baseline interview was 51.0% (68.2% in Arkhangelsk, 41.4% in Novosibirsk) of the total invitees. This reflects the proportion of all eligible participants of the relevant age and sex who were approached, invited, and agreed to participate in the study. After initial participation and interview, there was 96% health check attendance in Arkhangelsk and 83% in Novosibirsk, with an average 16-day gap to baseline interviews.

### 2.3. Data Collection

Systolic and diastolic blood pressure (SBP and DBP) were measured at the health check using an OMRON 705 IT automatic blood pressure monitor (OMRON Healthcare). The measurements were performed in a seated position three times with two-minute intervals between. The mean values of the second and third measurements were used in the analysis.

Blood samples were taken at the health check to assess cardiometabolic parameters, including total cholesterol (TC), low-density lipoprotein cholesterol (LDL-C), and glycated haemoglobin (HbA1c). Participants were asked to fast for at least four hours prior to the health check. The blood samples were aliquoted, frozen, and stored. Throughout the data collection, the samples were shipped on dry ice to a laboratory in Moscow and stored at −80°C. All laboratory analyses were performed in one batch at the end of the study. Levels of TC (mmol/L) and LDL-C (mmol/L) were assessed in blood serum using enzymatic color tests, and HbA1c (%) levels were assessed in the whole blood using immuno-turbidimetric tests (AU 680; Chemistry System Beckman Coulter).

Participants were requested to bring their current medications to the health check, and 27% of them did so. They were asked to show their currently used medications or if they did not bring them, to list them. Commercial names for up to seven medications per participant, as well as doses and frequencies were recorded and subsequently classified at the end of the study using the international WHO anatomical therapeutic chemical (ATC) classification system version 2016 [29].

Self-reports of hypertension, hypercholesterolemia, and DM were collected with three questions asked in the medical interview: “Have you ever been told by a doctor or nurse that you have…. high blood pressure?,” “high cholesterol?,” or “diabetes mellitus?” Responses like “do not know” were considered as negative answers.

### 2.4. Ascertaining the Presence of Risk Factors

Hypertension was defined as SBP >140 mmHg and/or DBP >90 mmHg at the health check and/or reported daily intake of antihypertensive medication (ATC classes C02, C03, C07, C08, or C09). Hypercholesterolemia was ascertained if a participant had total cholesterol ≥5.2 mmol/L and/or LDL cholesterol of >3.0 mmol/L and/or reported daily intake of lipid lowering medication (ATC class C10). Diabetes was defined as HbA1c ≥6.5% and/or self-reported the intake of antidiabetic medication (ATC class A10).

### 2.5. Assessing Awareness of Risk Factors

Awareness of hypertension, hypercholesterolemia, or DM was considered present if a participant had the condition(s) ascertained at the health check and self-reported positively to “have you ever been told by a doctor or nurse that you have” the conditions under investigation.

### 2.6. Associated Characteristics

To describe sociodemographic correlates of the awareness of risk factors, we used the following variables from baseline interview: age (years), sex (male or female), completed higher education (yes or no), and participant´s self-reported financial constrains indicating household income (low, middle, and high). Low income was defined as having financial difficulties to buy food or clothes; middle income—having enough money for food and clothes but experiencing constrains in buying large domestic appliances or a new car; high income—reporting no difficulties to buy a large new car but constraints to buy a flat or house, or no financial constraints at all.

Smoking data were recorded as self-reported daily smoking, and participants were divided into never, former, and current smokers. Data on alcohol consumption were collected using the Alcohol Use Disorders Identification Test (AUDIT) [30]. An AUDIT score ≥8 was defined as hazardous drinking.

Height (cm) was measured at the health check using the Seca® 217 stadiometer (Seca Ltd., Hamburg, Germany). Weight (kg) was measured using TANITA BC 418 body composition analyser (Tanita Corp., Tokyo, Japan). Body mass index (BMI) was calculated as weight in kilograms divided by squared height in meters. Obesity was defined as a BMI ≥30 kg/m^2^.

Data on the history of CVD diagnoses were obtained by asking the participants about having ever been diagnosed with angina, stroke, myocardial infarction, atrial fibrillation, or heart failure.

### 2.7. Sample

**Figure 1:**
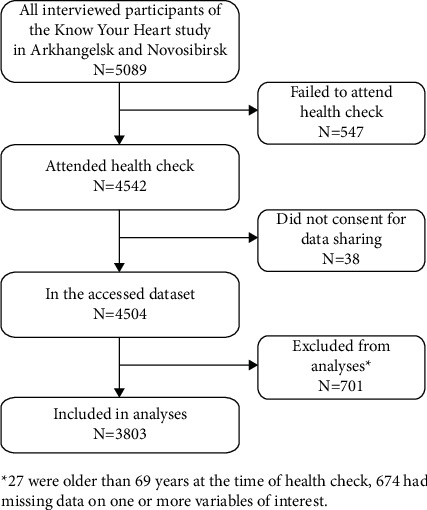
Flowchart describing the selection of study participants.

A total of 5089 men and women had the baseline interview as a part of the KYH study (Figure 1). Out of them, 547 failed to attend the health check, and 38 of the attendees did not provide the consent for sharing the collected data with third parties. Therefore, based on the data access application, the first author received the anonymized data of 4504 KYH participants, 2362 from Arkhangelsk and 2142 from Novosibirsk, limited to the variables used in this study. Another 701 participants were excluded from analyses for the following reasons: 27 were older than 69 years by the time of the health check, and 674 had missing or unusable data on one or more variables of interest. Correspondingly, 3803 KYH participants were included in the analyses. Participants with missing answers (*n* = 149) to the questions about ever being told by a doctor or nurse about having high blood pressure (*n* = 7), hypercholesterolemia (*n* = 114), or diabetes (*n* = 28) were treated as negative responses.

### 2.8. Statistical Analysis

The crude prevalence of hypertension, hypercholesterolemia, and DM, and the proportions of people aware of having these risk factors were presented with 95% confidence intervals (CI). Awareness estimates were also presented age- and sex-standardized [31]. Univariable and multivariable binary logistic regressions were used to assess associations between socio-demographic, lifestyle, and health characteristics with the awareness of each of the three risk factors. Finally, we estimated proportions (95% CI) of participants taking medication for any of the investigated three conditions, depending on their awareness of having these risk factors. Statistical analyses were performed using STATA V.17 (StataCorp, TX, USA).

### 2.9. Ethical Approval

The KYH study complied with the Declaration of Helsinki and was approved by the ethics committees of the London School of Hygiene & Tropical Medicine (approval number 8808, date 247/02/2015), Novosibirsk State Medical University (approval number 75, approval received 21/05/2015), the Institute of Preventative Medicine (no approval number; approval received 26/12/2014), Novosibirsk and the Northern State Medical University, Arkhangelsk (approval number 01/01-15, received 27/01/2015).

## 3. Results

The mean age of participants was 53.9 ± 9.7 years. The sample included 1512 residents of Novosibirsk and 2291 of Arkhangelsk. Fifty-eight percent had ascertained hypertension, 83.4% had hypercholesterolemia, and 8.7% had DM (Table 1). Age- and sex-standardized to ESP2013, the prevalence of hypertension, hypercholesterolemia, and DM were 53.5%, 81.6%, and 7.1%, respectively. There were no significant differences between Novosibirsk and Arkhangelsk (Table 3).

### 3.1. Awareness

Age- and sex-standardized prevalence of the awareness of hypertension was 79.3% among all participants with hypertension, significantly higher in Arkhangelsk (81.4%) compared to Novosibirsk (74.7%) (Table 2). The age-standardized prevalence was higher in females compared to males in the total sample (84.0% vs. 74.6%), and there were comparable differences between sexes in both sites taken separately.

The age- and sex-standardized prevalence of the awareness of hypercholesterolemia, the most prevalent of the three risk factors under study, was 44.7% among all participants with this risk factor. Like with hypertension, the awareness of hypercholesterolemia was higher in Arkhangelsk (46.5%) than in Novosibirsk (41.6%), and the age-standardized estimates were higher in females compared to males in the total sample (51.4% vs. 38.0%), with similar differences observed in both sites.

For DM, the least prevalent risk factor under study, age- and sex-standardized prevalence of the awareness in the total sample of diabetic participants was 61.2%. There were no significant differences between the sites in the total sample, but females with diabetes in Novosibirsk had substantially higher awareness prevalence (81.0%) compared to their Arkhangelsk counterparts (51.0%). Comparisons of the age-standardized estimates between males and females showed no differences.

### 3.2. Associated Characteristics

**Figure 2:**
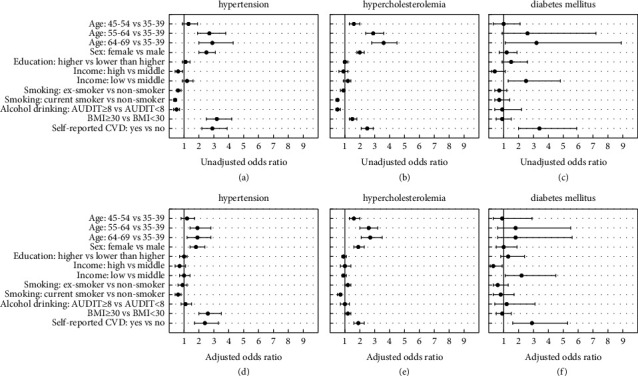
Adjusted and unadjusted odds ratios and confidence intervals for awareness of hypertension, hypercholesterolemia and diabetes mellitus by age, sex, education, income, smoking status, drinking status, BMI, and previous cvd experience. (a) Hypertension. (b) Hypercholesterolemia. (c) Diabetes mellitus. (d) Hypertension. (e) Hypercholesterolemia. (f) Diabetes mellitus.

In both univariable and multivariable regression analyses, the odds of being aware of having hypertension were higher among 55–69-year-old participants compared to 35–44-year-old participants, in females compared to males, in obese participants compared to the non-obese, and in those who self-reported CVD diagnoses compared to those who did not (Figure 2). High-income participants had reduced odds of awareness compared to those with middle incomes, but only in the univariate analysis. This was not observed after mutual adjustment for other confounding variables. Current smokers consistently demonstrated the reduced odds of the awareness of hypertension in both analyses.

Univariable and multivariable regressions showed increased odds of being aware of having hypercholesterolemia among those age 45–69 years compared to 35–44 years, in females compared to males, in obese compared to non-obese, and if having self-reported CVD diagnoses. Smokers and hazardous drinkers demonstrated the reduced odds of the awareness in univariable analyses, but only smoking sustained the association after all the covariates were mutually adjusted in the multivariable model.

The odds of the awareness of having DM were increased in participants with low income compared to the middle-income group in both univariable and multivariable analyses, while those in the high-income group had the reduced odds of DM only in the adjusted analysis. In both analyses, the odds of being aware were also increased in participants who self-reported CVDs compared to those who did not. The exact odds ratios with respective confidence intervals are provided in supplementary file 1.

### 3.3. Risk Factor Control by Awareness Status

**Figure 3:**
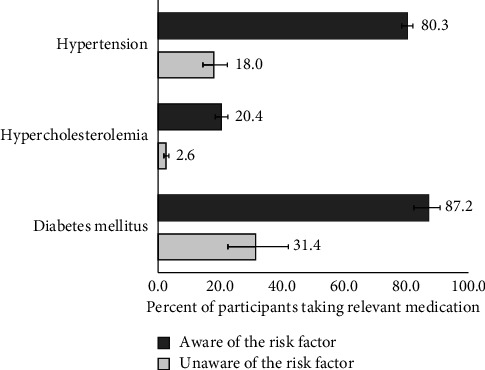
Proportions of participants taking medication for hypertension, hypercholesterolemia, and diabetes mellitus depending on the awareness of having these risk factors.

The proportion of participants with hypertension, hypercholesterolemia, and diabetes mellitus who self-reported relevant medication treatment was 71.1%, 11.6%, and 72.6%, respectively. These proportions showed disparities depending on participant´s awareness status. Among the participants who were aware of their condition, a substantially higher proportion received a relevant medication to control hypertension (80.3%), hypercholesterolemia (20.4%), and DM (87.2%) compared to participants who were unaware (18.0%, 2.6%, and 31.4%, respectively) (Figure 3).

## 4. Discussion

Our study demonstrated that the standardized prevalence estimates for awareness of hypertension, hypercholesterolemia, and DM in Russian adults with corresponding conditions were 79.3%, 44.7%, and 61.2%, respectively. Depending on the risk factor considered, the awareness had independent positive associations with older age, female sex, low income, obesity, prior CVD diagnoses, and negative association with current smoking.

### 4.1. Prevalence of Awareness

With standardization, the prevalence of hypertension was 53.5% in the study population, and the overall awareness proportion in participants with this risk factor (79.3%) was higher compared to the other two risk factors under study. This awareness proportion was comparable to the latest published estimates in Russia (68% in males, 86% in females) [3], and it was higher than the estimates for France at 37.5% [15], Luxemburg at 40% [32], Denmark at 40% [33], Portugal at 46% [34], Italy at 56% [35], Czech Republic at 67% [36], Spain at 64% [37], Sweden at 65% [38], Poland at 67% [39], and England at 65% [40]. However, awareness was lower than in the Netherlands at 80% [41], Germany at 80% [42], and Greece at 90% [43].

The standardized prevalence of hypercholesterolemia (81.6%) was the highest in the study population among the investigated risk factors, but the awareness proportion was the lowest (44.7%). At the same time, it was higher than the other estimates for Russia (11% in males, 21% in females) [3] and the findings in Luxembourg (15%) [32], comparable to the estimates for the Czech Republic (40%) [3] and Italy (43%) [35], but lower than for Poland (51%) [44].

DM had the lowest standardized prevalence (7.1%) among the three studied risk factors, with an awareness proportion of 61.2%. The latter was within the range of earlier published estimates for Russia varying from 31% and 45% for men and females, respectively [3], to the total estimate of 73% [45], and it was close to the proportion of diabetics treated with sugar-lowering drugs (59.3%) observed in Russian population sample in 2015–2018 [46]. The standardized DM awareness proportion in our study (61.2%) was also higher compared to Czech republic 54% [3], similar to Switzerland (65%) [47], Luxembourg (68%) [32] but lower than in Italy (77%) [48], Poland (77%) [3], and Portugal 87% [49].

Even though our awareness estimates of hypercholesterolemia and DM were low and our awareness of hypertension quite high, they are within the variability range observed in the countries with lower CVD mortality rates. However, our cross-country comparisons must be interpreted cautiously because of the comparability limitations. For example, different definitions of hypercholesterolemia (total cholesterol ≥4.9/5.0 and LDL ≥2.9) were used in the Czech Republic and Italy [3, 32, 35]. The participants in studies from Germany [42], Greece [43], and the Netherlands [41] were generally older, ranging from 55 to 95 years of age, compared to participants in our study who were 35–69 years of age. Contrary to our study, Italian estimates [35] disregarded the medication use of participants. Despite methodological differences, we believe in the reliability of the comparisons. Thus, low CVD awareness rates cannot solely explain the higher CVD mortality in Russia [6].

### 4.2. Factors Associated with Awareness

In agreement with previous research [17, 50–53], our study showed that older people were more likely to be aware of hypertension, hypercholesterolemia, and DM compared to younger people. The discrepancy might be attributable to the lack of healthcare utilization due to the overall low disease prevalence among the young and the correspondingly lower probability of exposure to the risk information [54]. Our findings of the higher awareness of hypertension and hypercholesterolemia in females compared to males are also similar to those of earlier studies [15, 19, 20, 55–59]. This might be explained by female's higher engagement with healthcare services and accordingly their increased likelihood of being informed about any health risks [15].

The associations observed between socioeconomic factors and the awareness of risk factors appear inconsistent with prior research. Contrary to earlier associations between high income and awareness [3, 16, 60], our study found higher awareness of DM only among low-income participants. Despite the existing evidence of the higher awareness levels among those with university education [16, 61–65] and also the opposite findings in a French study [15], we found no association between awareness and education. These findings may indicate specific features of the studied population, including the equal accessibility of the healthcare services, regardless of the socioeconomic status.

Other researchers have described the associations of the awareness of CVD risk factors with alcohol drinking [3, 19, 52, 60, 63, 66] and smoking [19, 56, 59, 67]. Our findings were similar with respect to smoking, but hazardous drinking was associated with the awareness of hypertension and hypercholesterolemia only before the adjustments for smoking and other covariates. This may be explained by a known association between smoking and hazardous drinking—the commonly cohabiting components of an unhealthy lifestyle. The adjustment for smoking may have excessively attenuated the association between the awareness and hazardous drinking. Based on this assumption, the overall negative effect of the unhealthy lifestyle on the awareness could be inferable.

With respect to obesity and previous cardiovascular events, our findings agree with the existing evidence that living with these conditions increases awareness of hypertension, hypercholesterolemia, and DM [15, 18, 20, 64, 68–70], which may be explained by higher health concerns and frequent health care contacts.

Finally, our study has demonstrated that those aware of their risk factors were more likely to be receiving proper medication compared to those unaware. Notably, our findings reveal a disparity between the awareness rates (44.7%) and the treatment rates for hypercholesterolemia, which were relatively low (11.6%). Conversely, participants with hypertension and diabetes mellitus (DM) displayed comparatively higher rates of treatment. Despite our identification of low treatment rates for hypercholesterolemia and relatively favorable treatment rates for hypertension, prior studies report extremely low antihypertensive and lipid-lowering medication adherence in Russia [71]. On the other hand, self-medication for high blood pressure without having hypertension diagnosed by a doctor is an acknowledged problem in Russia because of the non-prescription sales of antihypertensives [71]. As for antidiabetics, their intake by those not aware of having DM might be explained by the common prescription of glucose-lowering medication to those at the so-called prediabetes stage [72]. The role of awareness in a Russian setting is therefore crucial not only in recognising the risk of hypertension, hypercholesterolemia, and DM but also in treatment, adherence, control, and overall prevention of unnecessary self-medication.

### 4.3. Strengths and Limitations

A strength of this study is that we investigated awareness of three major CVD risk factors using data from a large population-based sample of Russian adults, where self-reports could be assessed against the objective presence of the same risk factors, as defined by examinations performed by health professionals. However, the relatively low prevalence of DM resulted in a rather low precision of the awareness estimates and the power to identify the associated characteristics.

An important limitation is that the KYH sample is comprised of only urban residents of two Russian cities, which may not be representative of the Russian population overall. Urban residents have better access to healthcare, which might lead to an overestimation of the presented awareness proportions [3]. Therefore, the generalizability of the findings is limited to the Russian urban population.

The overall response proportion in the KYH was relatively low and varied between the cities 68% in Arkhangelsk and 41% in Novosibirsk [28]. We assessed nonresponse bias by comparing the risk factors and the awareness proportions between the sites which resulted in minor differences (Table 3) indirectly indicating that selection bias was not substantial. However, assessments do not exclude the possibility of selection bias for the whole. KYH was presented as a study focused on cardiovascular health, and people more concerned with their health may have been more likely to participate in it.

The ascertainment of the risk factors included using the self-reported data on medication use, which could limit the objectivity. However, we believe the bias could not be large since participants were asked to show their regularly taken medications or to list their commercial names, doses, and frequencies. Antihypertensives, lipid-lowering drugs, and antidiabetics require long-time daily intake, so failure to remember their names was unlikely with adherence. In addition, several studies show a good agreement between self-reported CVD medication and pharmacy records [73, 74].

## 5. Conclusion

Awareness of hypertension is relatively high, but for high cholesterol and diabetes mellitus awareness is relatively low. Lower awareness may lead to lower levels of preventative treatment for key CVD risk factors which contribute to higher CVD morbidity and mortality in Russia. We observed that awareness levels varied by age, sex, income, smoking status, obesity, and comorbidities. These findings may guide targeted awareness-raising interventions.

## Figures and Tables

**Table 1:** Characteristics of the total study population and stratified by sex.

Characteristics	Total sample *N* = 3803	Males *N* = 1591	Females *N* = 2212
	Abs (%)		
City of residence			
Arkhangelsk	2291 (60.2)	957 (60.1)	1334 (60.3)
Novosibirsk	1512 (39.8)	634 (39.9)	878 (39.7)
Age (years)			
35–44	825 (21.7)	314 (19.7)	511 (23.1)
45–54	1051 (27.6)	451 (28.4)	600 (27.1)
55–64	1251 (32.9)	542 (34.0)	709 (32.1)
65–69	676 (17.8)	284 (17.9)	392 (17.7)
Higher (university) education	1430 (37.6)	563 (35.4)	867 (39.2)
Household income^a^			
Low	720 (18.9)	263 (16.5)	457 (20.7)
Middle	2862 (75.3)	1219 (76.6)	1643 (74.3)
High	221 (5.8)	109 (6.9)	112 (5.0)
Smoking status			
Never	1886 (49.6)	404 (25.4)	1482 (67.0)
Former	951 (25.0)	586 (36.8)	365 (16.5)
Current	966 (25.4)	601 (37.8)	365 (16.5)
Hazardous drinking^b^	435 (11.4)	393 (24.7)	42 (1.9)
Obesity, BMI ≥30	1207 (31.7)	417 (26.2)	790 (35.7)
Self-reported CVD^c^	941 (24.7)	379 (23.8)	562 (25.4)
Ascertained risk factors^d^			
Hypertension	2206 (58.0)	1017 (63.9)	1189 (53.8)
Hypercholesterolemia	3171 (83.4)	1325 (83.3)	1846 (83.5)
Diabetes mellitus	329 (8.7)	122 (7.7)	207 (9.4)
Self-reported risk-factors			
Hypertension	2224 (58.5)	914 (57.5)	1310 (59.2)
Hypercholesterolemia	1686 (44.3)	563 (35.4)	1123 (50.8)
Diabetes mellitus	298 (7.8)	105 (6.6)	193 (8.7)
Medication use			
Antihypertensives^e^	1547 (40.7)	590 (37.1)	957 (43.3)
Lipid modifying agents^f^	367 (9.6)	162 (10.2)	205 (9.3)
Antidiabetics^g^	239 (6.3)	74 (4.5)	165 (7.5)

^a^Low income was defined as difficulties to buy food or clothes, middle–large domestic appliances or new car, high-flat, house, or having no financial constraints; ^b^Alcohol Use Disorders Identification Test (AUDIT) score ≥8; ^c^Ever diagnosed with angina, stroke, myocardial infarction, atrial fibrillation, or heart failure; ^d^Ascertained at the health check by objective measurements and/or data on medication use; ^e^Codes C02, C03, C07, C08, C09 according to WHO anatomical therapeutic chemical classification (ATC); ^f^Code C10 according to ATC; ^g^Code A10 according to ATC.

**Table 2:** Prevalence of the awareness of hypertension, hypercholesterolemia, and diabetes mellitus among study participants with corresponding risk factors in the total sample and with stratification by study site and sex.

	Total study population			Novosibirsk study population			Arkhangelsk study population		
	*N* ^a^	Crude estimates	Standardized estimates^b^	*N* ^a^	Crude estimates	Standardized estimates^a^	*N* ^a^	Crude estimates	Standardized estimates^a^
		Percent (95% CI)			Percent (95% CI)			Percent (95% CI)	
*Total sample*									
Hypertension	2206	83.6 (82.0; 85.1)	79.3 (76.9; 81.5)	890	81.4 (78.7; 83.8)	74.7 (70.4; 78.5)	1316	85.2 (83.2; 87.0)	81.4 (78.4; 84.0)^†^
Hypercholesterolemia	3171	50.3 (48.6; 52.1)	44.7 (42.9; 46.5)	1288	49.4 (46.7; 52.1)	41.6 (38.9; 44.4)	1883	51.0 (48.7; 53.2)	46.5 (44.2; 48.8)^†^
Diabetes mellitus	329	73.9 (68.8; 78.3)	61.2 (53.4; 68.4)	148	77.0 (69.5; 83.1)	69.1 (60.5; 76.5)	181	71.3 (64.2; 77.4)	58.3 (50.2; 66.1)

*Males*									
Hypertension	1017	77.1 (74.4; 79.6)	74.6 (71.2; 77.7)	396	72.0 (67.3; 76.2)	65.5 (58.9; 71.5)	621	80.4 (77.0; 83.3)	79.0 (75.0; 82.4)^†^
Hypercholesteremia	1325	40.2 (37.5; 42.8)	38.0 (35.4; 40.8)	532	38.5 (34.5; 42.8)	34.0 (29.9; 38.3)	793	41.2 (37.9; 44.7)	40.3 (36.8; 43.8)
Diabetes mellitus	122	72.1 (63.4; 79.4)	60.5 (51.1; 69.1)	58	70.7 (57.7; 81.0)	57.2 (43.5; 69.9)	64	73.4 (61.2; 82.9)	65.7 (55.2; 74.9)

*Females*									
Hypertension	1189	89.2 (87.3; 90.9)	84.0 (80.6; 86.9)^‡^	494	88.9 (85.8; 91.4)	83.8 (78.2; 88.3)^‡^	695	89.5 (87.0; 91.6)	83.8 (79.2; 87.5)^‡^
Hypercholesterolemia	1846	57.6 (55.4; 59.9)	51.4 (49.0; 53.7)^‡^	756	57.0 (53.5; 60.5)	49.3 (45.7; 52.9)^‡^	1090	58.1 (55.1; 61.0)	52.7 (49.7; 55.7)^‡^
Diabetes mellitus	207	74.9 (68.5; 80.4)	61.8 (49.3; 73.0)	90	81.1 (71.6; 88.0)	81.0 (70.4; 88.4)	117	70.1 (61.1; 77.7)	51.0 (38.5; 63.3)^†^

^a^Number of study participants with objectively ascertained risk factor. ^b^Age- and sex-standardized to ESP2013 for the total sample, age-standardized to ESP2013 for males and females. ^†^*p* < 0.05 for difference between sites, binary logistic regression with adjustments for age for males and females and also for sex for the total sample. ^‡^*p* < 0.05 for difference between sexes, logistic regression with adjustments for.

**Table 3:** Prevalence of hypertension, hypercholesterolemia, and diabetes mellitus in the total study population (*N* = 3803) and by study site (Novosibirsk, *N* = 1512; Arkhangelsk, *N* = 2291).

	Crude estimates	Standardized estimates^a^
	Percent (95% CI)	
*Total sample*		
Hypertension	58.0 (56.4; 59.6)	53.5 (52.0; 55.1)
Hypercholesterolemia	83.4 (82.2; 84.5)	81.6 (80.3; 82.9)
Diabetes mellitus	8.7 (7.8; 9.6)	7.1 (6.4; 7.9)

*Novosibirsk*		
Hypertension	58.9 (56.4; 61.3)	52.2 (49.6; 54.8)
Hypercholesteremia	85.2 (83.3; 86.9)	82.8 (80.6; 84.9)
Diabetes mellitus	9.8 (8.4; 11.4)	7.9 (6.6; 9.3)

*Arkhangelsk*		
Hypertension	57.4 (55.4; 59.5)	54.4 (52.5; 56.4)
Hypercholesterolemia	82.2 (80.6; 83.7)	80.9 (79.1; 82.5)
Diabetes mellitus	7.9 (6.9; 9.1)	6.7 (5.8; 7.7)

^a^Age- and sex-standardized to European standard population 2013 (ESP2013).

## Data Availability

The data used to support the findings of this study may be released upon application on the website https://metadata.knowyourheart.science after contacting David Leon at david.leon@lshtm.ac.uk.

## Supplementary Materials

Supplementary Materials Supplementary file 1: adjusted and unadjusted odds ratios and confidence intervals for awareness of hypertension, hypercholesterolemia, and diabetes mellitus by age, sex, education, income, smoking status, drinking status, BMI, and previous cvd experience.
