# Supplementary material for: Awareness of Hypertension, Hypercholesterolemia, and Diabetes Mellitus and Associated Characteristics in Russian Adults
Source: Int J Hypertens. 2024 Mar 25;2024:8542671. doi: 10.1155/2024/8542671 (PMC10985646; doi:10.1155/2024/8542671)
Supplement: Supplementary Materials — Supplementary file 1: adjusted and unadjusted odds ratios and confidence intervals for awareness of hypertension, hypercholesterolemia, and diabetes mellitus by age, sex, education, income, smoking status, drinking status, BMI, and previous cvd experience. [file 8542671.f1.docx]

**Supplementary file 1:** Participants’ characteristics associated with the awareness of having hypertension, hypercholesterolemia, and diabetes.

| **Characteristics** | **Awareness of hypertension**  N=2206 | | **Awareness of hypercholesterolemia**  N=3171 | | **Awareness of diabetes mellitus**  N=329 | |
| --- | --- | --- | --- | --- | --- | --- |
|  | **OR**  **(95% CI) ^a^** | **OR_adj_**  **(95% CI) ^b^** | **OR**  **(95% CI) ^a^** | **OR_adj_**  **(95% CI) ^b^** | **OR**  **(95% CI) ^a^** | **OR_adj_**  **(95% CI) ^b^** |
| **Age, years** |  | |  | |  | |
| - 35-44 | 1.0 |  | 1.0 |  | 1.0 |  |
| - 45-54 | 1.3 (0.9; 1.9) | 1.2 (0.8; 1.7) | **1.6 (1.3; 2.0)** | **1.6 (1.3; 2.0)** | 1.0 (0.3; 3.1) | 0.9 (0.3; 2.9) |
| - 55-64 | **2.7 (1.9; 3.8)** | **1.9 (1.4; 2.8)** | **2.9 (2.4; 3.6)** | **2.6 (2.0; 3.2)** | 2.6 (0.9; 7.2) | 1.8 (0.6; 5.5) |
| - 65-69 | **2.9 (2.0; 4.3)** | **1.9 (1.2; 2.8)** | **3.6 (2.8; 4.5)** | **2.7 (2.1; 3.5)** | **3.2 (1.1; 8.9)** | 1.8 (0.6; 5.6) |
| **Sex** |  |  |  |  |  |  |
| - Male | 1.0 |  | 1.0 |  | 1.0 |  |
| - Female | **2.5 (2.0; 3.1)** | **1.8 (1.4; 2.4)** | **2.0 (1.8; 2.3)** | **1.9 (1.6; 2.3)** | 1.2 (0.7; 1.9) | 1.0 (0.5; 1.9) |
| **Higher education** | | |  | |  | |
| - Yes | 1.0 |  | 1.0 |  | 1.0 |  |
| - No | 1.1 (0.9; 1.4) | 1.0 (0.7; 1.2) | 1.0 (0.9; 1.2) | 0.9 (0.8; 1.1) | 1.5 (0.9; 2.6) | 1.3 (0.8; 2.4) |
| **Income** ^c^ |  | |  | |  | |
| - Middle | 1.0 |  | 1.0 |  | 1.0 |  |
| - High | **0.6 (0.4; 0.9)** | 0.7 (0.4; 1.1) | 0.9 (0.6; 1.2) | 1.0 (0.7; 1.4) | 0.4 (0.2; 1.1) | **0.3 (0.1; 0.9)** |
| - Low | 1.2 (0.9; 1.6) | 1.0 (0.7; 1.4) | 1.2 (0.9; 1.4) | 0.9 (0.8; 1.1) | **2.5 (1.3; 4.8)** | **2.2 (1.1; 4.5)** |
| **Smoking** |  | |  | |  | |
| - Never | 1.0 |  | 1.0 |  | 1.0 |  |
| - Former | **0.6 (0.5; 0.8)** | 0.9 (0.6; 1.2) | 0.9 (0.7; 1.0) | 1.2 (1.0; 1.4) | 0.7 (0.4; 1.2) | 0.6 (0.3; 1.3) |
| - Current | **0.4 (0.3; 0.5)** | **0.6 (0.4; 0.8)** | **0.5 (0.4; 0.5)** | **0.7 (0.5; 0.8)** | 0.7 (0.4; 1.4) | 0.8 (0.3; 1.7) |
| **Hazardous drinking** ^d^ | | |  | |  | |
| - No | 1.0 |  | 1.0 |  | 1.0 |  |
| - Yes | **0.5 (0.3; 0.7)** | 1.1 (0.8; 1.5) | **0.5 (0.4; 0.7)** | 1.0 (0.7; 1.3) | 0.9 (0.4; 2.2) | 1.2 (0.4; 3.1) |
| **Obesity, BMI ≥30** | | |  | |  | |
| - No | 1.0 |  | 1.0 |  | 1.0 |  |
| - Yes | **3.2 (2.5; 4.2)** | **2.6 (2.0; 3.5)** | **1.5 (1.3; 1.8)** | **1.2 (1.0; 1.4)** | 0.9 (0.5; 1.5) | 0.9 (0.5; 1.5) |
| **Self-reported CVDs** ^e^ | | |  | |  | |
| - No | 1.0 |  | 1.0 |  | 1.0 |  |
| - Yes | **2.9 (2.2; 3.9)** | **2.4 (1.7; 3.3)** | **2.5 (2.1; 2.9)** | **1.9 (1.6; 2.3)** | **3.4 (2.0; 5.9)** | **2.9 (1.6; 5.3)** |

Significant odds ratios (p<0,05) are highlighted in bold. ^a^ Univariable logistic regression model; ^b^ Multivariable logistic regression model with all covariates in the table mutually adjusted; ^c^ Low income – difficulties to buy food or clothes, middle – to buy large domestic appliances or new car, high – to buy flat, house, or having no financial constraints; ^d^ Alcohol Use Disorders Identification Test (AUDIT) score ≥ 8; ^e^ Ever diagnosed with angina, stroke, myocardial infarction, atrial fibrillation, or heart failure.
